# Stability of Phenolic Compounds, Antioxidant Activity and Color Parameters in Colored-Flesh Potato Chips

**DOI:** 10.3390/molecules28166047

**Published:** 2023-08-14

**Authors:** Catalina Bravo, Fabiola Peña, Javiera Nahuelcura, Catalina Vidal, Felipe González, Felipe Jiménez-Aspee, Luis Bustamante, Boris Contreras, Antonieta Ruiz

**Affiliations:** 1 Departamento de Ciencias Químicas y Recursos Naturales, Scientific and Technological Bioresource Nucleus BIOREN-UFRO, Universidad de La Frontera, Temuco 4811230, Chile; 2 Programa de Doctorado en Ciencias Agroalimentarias y Medioambiente, Facultad de Ciencias Agropecuarias y Forestales, Universidad de La Frontera, Temuco 4811230, Chile; 3 Programa de Doctorado en Ciencias Mención Biología Celular y Molecular Aplicada, Facultad de Ciencias Agropecuarias y Forestales, Universidad de La Frontera, Temuco 4811230, Chile; 4 Department of Food Biofunctionality (140b), Institute of Nutritional Sciences, University of Hohenheim, Garbenstr. 28, D-70599 Stuttgart, Germany; 5 Departamento de Análisis Instrumental, Facultad de Farmacia, Universidad de Concepción, Concepción 4030000, Chile; 6 C-M SpA, Loteo Pozo de Ripio s/n, Parque Ivian II, Puerto Varas 5550000, Chile

**Keywords:** colored-flesh potato, anthocyanin, hydroxycinnamic acid, antioxidant activity, HPLC-DAD-ESI-MS/MS

## Abstract

Potato (*Solanum tuberosum*) chips are the most consumed snacks worldwide today. Colored potato chips prepared from potato cultivars with red and purple flesh are a novel alternative to traditional potato chips because of their higher phenolic compound content, such as anthocyanins and hydroxycinnamic acid derivatives (HCADs), which might make these chips healthier compared with traditional chips. There is little information on the stability of these compounds. In this study, the nutritional value of these chips was evaluated by determining phenolic profiles, antioxidant activity and color parameters with liquid chromatography diode array and mass spectrometry detection (HPLC-DAD-ESI-MS/MS) and spectrophotometric methods during storage for four months. Five anthocyanins and three HCADs were detected, with the latter compounds being the most abundant, with concentrations on average between the first (97.82 mg kg^−1^) and the last (31.44 mg kg^−1^) week of storage. Similar trends were observed in antioxidant activity and stability, with the CUPRAC method showing the highest response among all the methods employed. The color indices were stable throughout the storage time. Based on these results, colored-flesh potato chips are an optimal alternative for consumption because of their high retention of phenolic compounds and antioxidant activity during storage, providing potential benefits to human health.

## 1. Introduction

Potato (*Solanum tuberosum* sp. tuberosum) is the fourth most important crop in the world in terms of human food after maize, wheat and rice [1]. Potato tubers are rich in vitamins such as ascorbic acid, B1, B3, folate, riboflavin and pantothenic acid and contain minerals such as potassium, magnesium and phosphorus, as well as bioactive molecules such as carotenoids and anthocyanins [2]. Southern Chile provides a great diversity of potato genotypes that present high phenolic compound contents in the pulp and peel, including anthocyanins and hydroxycinnamic acid derivatives (HCAD) [3,4].

Anthocyanins display numerous potential health benefits, and they have been investigated recently for their use as possible clinical treatments for many human disorders [5]. They have both anti-inflammatory and antioxidant properties, which have demonstrated effectiveness in different in vivo and in vitro models of several chronic conditions, such as cardiovascular disease, ophthalmic disorders, obesity, diabetes and different types of cancer [6,7,8]. Similarly, HCADs are of high importance because of their health-benefiting effects. HCADs are mainly recognized as potent antioxidants that are involved in the prevention of several diseases connected to oxidative stress, such as cardiovascular and neurodegenerative diseases, as well as certain types of cancer [9,10,11].

The potato chip industry is one of the most important and widespread food industries. Potato chips are the most consumed snack worldwide today, with an expected growth rate of USD 1.7 million in revenue, which is projected to reach an increase of 4.4% in 2025 [12]. Colored potato chips prepared from potato cultivars with red and purple flesh are a novel alternative to traditional potato chips. Given the higher phenolic compound content, such as anthocyanins, in red- and purple-fleshed potato chips relative to traditional potato chips, these chips may be healthier [3,13]. On the other hand, colored potatoes also contain high concentrations of potassium, as well as other minerals like zinc, iron, copper, manganese, calcium and magnesium [14,15]. With regard to sugar composition, potatoes are a major source of carbohydrates, which are mainly present as starch. Studies have reported the presence of sucralose, glucose, fructose and myoinositol in different colored potato genotypes [14].

The stability of anthocyanins is highly affected by the potato growth environment and other factors, such as light, pH, temperature, oxygen, metal ions and enzymes [4]. Unlike anthocyanins, in which changes after processing have been studied in detail [16], there is thus far little information available about the effect of processing on HCAD profiles and concentrations.

A recent study by Tajner-Czopek et al. [17] revealed the effect of the various production stages of potato chips made from potatoes of different flesh colors on the content of hydroxycinnamic acids. The results showed that, after peeling, colored potatoes, mainly purple potatoes, had more residual hydroxycinnamic acids (5-*O*-caffeoylquinic acid, 4-*O*-caffeoylquinic acid, 3-*O*-caffeoylquinic acid and 3,4-dihydroxycinnamic acid) in the pulp relative to light-yellow potato varieties. Furthermore, it was observed that the pre-drying and frying stages resulted in the highest losses of the determined hydroxycinnamic acids, at 91% and 97%, respectively. Stability studies of colored potato extracts carried out by Ercoli et al. [18] showed a decrease in the concentration of anthocyanins. In contrast, the total antioxidant activity of liquid extracts remained constant throughout the study. Ruiz et al. [3] also reported a decrease in the total anthocyanin concentrations after cooking or frying processes. Conversely, an increase in HCADs in potato samples after cooking and frying compared with fresh samples has also been detected [3,4]. On the other hand, Ercoli et al. [4] reported a decrease in total phenols in potatoes after cooking. In contrast, Ruiz et al. [3] observed a higher increase in total phenolics after frying. Regarding antioxidant activity, studies have reported an increase after cooking and also after frying [3,4].

According to these preceding studies, the general aim of this study is to evaluate the nutritional value of colored potato chips by determining their phenolic profiles and antioxidant activity.

## 2. Results

### 2.1. Identification and Quantification of Anthocyanins and HCADs

A total of five different anthocyanins were detected in the samples, whereas, for HCADs, two compounds were detected up to week 12, but from this point onward, a third HCAD was detected (Table 1, Figures S1 and S2).

Quantifications of the anthocyanins were carried out using external calibration with petunidin-3-glucoside (P3G) as a standard (Table 2 and Table 3). Among the anthocyanins identified, petunidin-3-feruloylrutinoside-5-glucoside presented the highest concentration in the initial week of analysis, reaching an average of 3.24 ± 0.94 mg kg^−1^, and it was not detected (nd) in the last week of storage. In contrast, peonidin-3-p-coumaroylrutinoside-5-glucoside and an unidentified compound (peak 8) showed the lowest values, and peonidin-3-p-coumaroylrutinoside-5-glucoside reached the highest concentration of 1.21 ± 0.24 mg kg^−1^ at week 18 of analysis, but with no significant differences between the initial and final conditions. The quantification of HCADs was carried out using an external calibration curve using chlorogenic acid (CQA) as a standard. The compound with the highest values corresponded to 5-caffeoylquinic acid with an initial concentration of 97.82 ± 34.47 mg kg^−1^ and a concentration of 26.08 ± 8.20 mg kg^−1^ in the last week of analysis; in contrast, the compound with the lowest values corresponded to the caffeoylquinic acid isomer, with concentrations ranging from 1.79 to 1.33 mg kg^−1^ between the initial and final determinations (Table 2).

**Figure 1:**
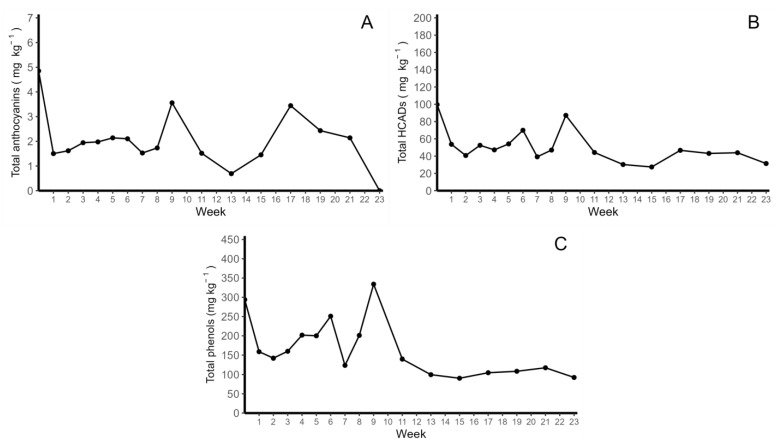
Stability of colored-flesh potato chips in storage for four months. (**A**) total anthocyanin concentrations found using HPLC-DAD; (**B**) total HCAD concentrations found using HPLC-DAD; and (**C**) total phenols concentrations determined using the Folin–Ciocalteu method.

The average concentrations of total anthocyanins and total HCADs were initially 4.85 mg kg^−1^ and 99.60 mg kg^−1^, respectively. At the end of week 2, decreases of 69.02% and 46.08% for each group were observed. The anthocyanin content varied throughout the experiment, reaching the lowest point after 23 weeks of storage, with contents below the quantification limit of our method (Figure 1A). In contrast, the HCADs were more stable over time, reaching a final concentration of 31.44 mg kg^−1^ at the end of week 23 (Figure 1B).

The stability of total phenols was determined via the quantification of their content at different time points using the Folin–Ciocalteu detection method with gallic acid (GA) as the standard (Figure 1C). The initial concentration was, on average, 293.90 ± 80.29 GA equivalents kg^−1^, while the concentration in the last week of storage was 92.42 ± 30.46 mg GA equivalents kg^−1^. Initially, a decay of 54.02% in the total phenol concentration was observed, followed by stabilization during the storage period. The sum of the total anthocyanins and HCADs ranged between 1.44% and 26.90% of the total phenols detected in the fried potato chips.

### 2.2. Determination of Antioxidant Activity

**Figure 2:**
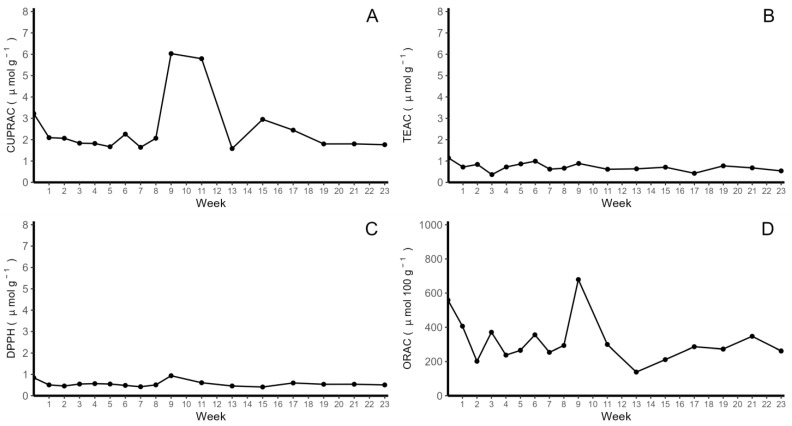
Stability of colored-flesh potato chips in storage for four months. (**A**) antioxidant activity determined using the cupric reducing antioxidant capacity (CUPRAC) method, (**B**) antioxidant activity determined using the Trolox equivalent antioxidant capacity (TEAC) method, (**C**) antioxidant activity determined using the free radical 2,2-diphenyl-1-picrylhydrazyl (DPPH) method and (**D**) antioxidant activity determined using oxygen radical absorbance capacity (ORAC) method.

The antioxidant activity of colored-flesh potato chips was determined by using the Trolox equivalent antioxidant capacity (TEAC), 2,2-diphenyl-1-picrylhydrazyl (DPPH), cupric reducing antioxidant capacity (CUPRAC) and oxygen radical absorbance capacity (ORAC) methods. The highest level of antioxidant activity was found with the CUPRAC method (Figure 2A), reaching an initial concentration of 3.22 µmol Trolox equivalents g^−1^, followed by a decrease of 35.02% at the end of week 2, and then, stable values were observed until week 9. Then, an increase in activity was observed between weeks 9 and 12, with a subsequent decrease to values close to 2 µmol Trolox g^−1^. In contrast, the TEAC assay showed stable antioxidant activity throughout the storage period, with a 52.94% decrease in the last week compared with the initial measurements (1.14 µmol g^−1^) (Figure 2B). In the case of the DPPH method (Figure 2C), results similar to those of the TEAC method were observed, with an initial concentration of 0.84 µmol Trolox equivalents g^−1^ and an average decrease of 39.64% in antioxidant activity after the second week, followed by stable activity throughout the storage period. With the ORAC method (Figure 2D), the initial value was, on average, 558.79 µmol Trolox equivalents 100 g^−1^, with high variability throughout the storage period.

### 2.3. Color Determinations

**Figure 3:**
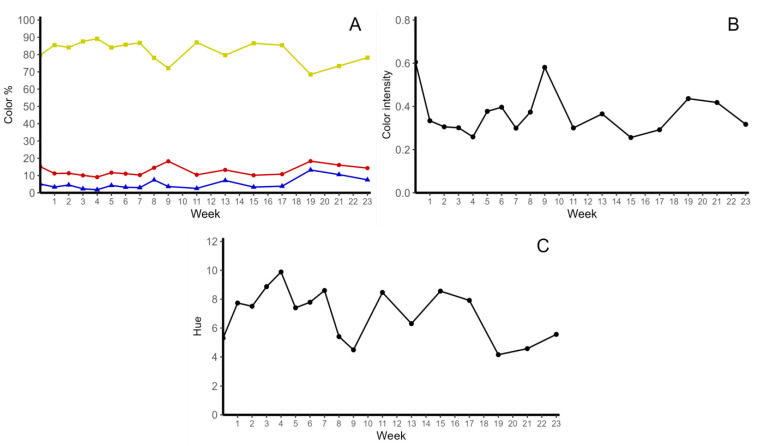
Stability of color parameters in colored-flesh potato chips in storage for four months. (**A**) percentage of yellow, red and blue colors; (**B**) color intensity; (**C**) hue.

Yellow, red and blue colorations were evaluated in the potato chips. The initial conditions of the potato chips represented, on average, 79.82% yellow, 15.98% red and 5.08% blue coloration. The proportion of each color remained stable during the entire storage period (Figure 3A). For the color intensity, the initial value was found to be 0.60, but this value decreased to 0.32 in the last week of analysis. Moreover, the initial hue value was determined to be 5.30, followed by a distribution of values during the storage period, but no significant differences were detected (Figure 3B,C).

### 2.4. Principal Component Analysis of Initial and Final Conditions

**Figure 4:**
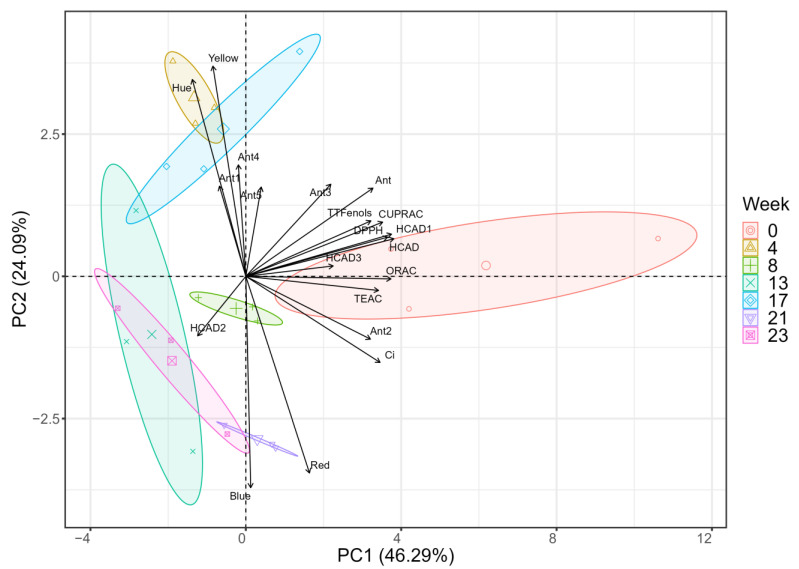
Principal component analysis (PCA) for the colored-flesh potato chips samples at 0, 4, 8, 13, 17, 21 and 23 weeks of storage. Percentage values in parentheses after PC1 and PC2 indicate the experimental variations explained by each component. Ant1, Ant2, Ant3, Ant4 and Ant5: quantified anthocyanins; Ant: total anthocyanins; HCAD1, HCAD2 and HCAD3: quantified hydroxycinnamic acids; HCAD: total hydroxycinnamic acids; TTFenols: total phenolics; TEAC: Trolox equivalent antioxidant capacity; CUPRAC: cupric reducing antioxidant capacity; DPPH: free-radical 2,2-diphenyl-1-picrilhidrazil; ORAC: oxygen radical absorbance capacity. Color intensity (Ci), hue, yellow, red and blue are the color parameters analyzed here.

Principal component analysis (PCA) was carried out considering all the evaluated parameters (Figure 4). This analysis revealed the formation of four distinct groups based on the distribution of experimental variables in the two principal components (PCs). Specifically, PC1 accounted for 46.29% and PC2 accounted for 24.09% of the total experimental variance (Figure 4). PC1 showed a positive correlation with red color; antioxidant activities (DPPH, CUPRAC, TEAC and ORAC); total hydroxycinnamic acids; the identified hydroxycinnamic acids 1–3; total phenols; color intensity; total anthocyanins; blue color; and anthocyanins 2, 3 and 5. On the other hand, it showed a negative correlation with yellow color, hue, hydroxycinnamic acid 2, anthocyanin 4 and anthocyanin 1. PC2 was also positively related to antioxidant activities (DPPH and CUPRAC); hydroxycinnamic acids 2 and 3; red color; color intensity; and anthocyanins 1, 4 and 5. The analyses in this study lasted twenty-four weeks because this time span was within the optimal period for potato chip consumption, as indicated by the expiration date. During this period, the results obtained showed stability until weeks 8 and 17 of storage, after which, a noticeable change in the phenolic profile, antioxidant activity and color parameters was observed during storage (Figure 1, Figure 2 and Figure 3). In this sense, PCA showed a correlation between the variable red color, antioxidant activities, total hydroxycinnamic acids, hydroxycinnamic acids 1–3, total phenols, color intensity, total anthocyanins and anthocyanins 2 and 3. Furthermore, hue, yellow color and anthocyanins 1 and 4 were associated with a distinct group at weeks 4 and 17. Moreover, HCAD 2 and blue color were associated with weeks 13 and 23. In this study, the most important variables are the antioxidant activities determined using the DPPH, CUPRAC and TEAC methods, as well as the intensity, red coloration, total phenols, anthocyanins and most of the HCADs detected. A high correlation between anthocyanins and most of the HCADs detected was related to the samples from the initial week, and a correlation between phenolic compounds, antioxidant activity testing and intensity could also be observed. On the other hand, after twenty-four weeks of storage, the variables mentioned above were less important because of the loss of stability observed over time (Figure 1 and Figure 2). HCAD 2, hue and yellow coloration were more related to the samples from the last week of the study.

## 3. Discussion

In other studies on colored-flesh potatoes, after the cooking and frying processes, a variety of compounds were identified [3,4]. In the case of anthocyanins, the most abundant compounds were petunidin, malvidin and pelargonidin derivatives, such as petunidin-3-(p-coumaroyl)-rutinoside-5-glucoside, malvidin-3-(p-coumaroyl)-rutinoside-5-glucoside and pelargonidin-3-(p-coumaroyl)-rutinoside-5-glucoside, among others, of which petunidin derivatives were more abundant in purple-fleshed potato genotypes (CB2011.189), and pelargonidin derivatives were prominent in red-fleshed potato genotypes (CR2002.8) [4]. For HCADs, trans 3-, 4- and 5-caffeoylquinic acids were detected, with 5-caffeoylquinic acid being most abundant in both cooked and fried potatoes [3,4,16].

In the present study, total HCAD concentrations were higher than the total anthocyanin concentrations. A similar finding was reported by Ruiz et al. [3], who showed that the total HCAD concentrations determined in fried potato chips were ten times higher than the total anthocyanin concentrations. The decrease in anthocyanin and HCAD concentrations can be explained by several factors. High temperatures can induce the thermal degradation of anthocyanins following the hydrolytic cleavage of glycosidic bonds and/or the cleavage of the flavonoid core. This degradation produces a decrease in anthocyanin concentrations in foods during cooking or storage, and degradation is accelerated by the presence of oxygen and light [18,19,20,21]. Regarding the effects on HCADs, although there is little information available about their profiles and concentrations, previous studies have reported high stability in these compounds at different temperature and pH conditions in dyes extracted from colored-flesh potatoes [18,22]. Similarly, Ruiz et al. [3] observed a higher increase in HCADs after frying compared with concentrations in fresh potato samples. Additionally, it has been reported that UV radiation can generate trans-to-cis HCAD isomerization [23].

Comparing the stability of anthocyanins from colored potato chips with other sources of anthocyanins could be important in the food industry. For example, it has been reported that the stability of anthocyanins from strawberry nectars is quite low after forty-two days of storage [24]. One factor that may cause this low stability is the presence of ascorbic acid. The addition of ascorbic acid to various fruit juices for enrichment was found to accelerate the loss of anthocyanins and polymeric color formation. Moreover, as the concentration of ascorbic acid increased from 60 to 80 mg L^−1^, anthocyanin degradation rates in sour cherry and pomegranate juices increased significantly [25,26]. Similarly, red raspberry juice anthocyanins were unstable during thirty days of storage at 37 °C, showing a decrease in the total anthocyanin concentration of 90% [27]. The difference in the stability of anthocyanins from food sources is related to various factors, including their structure. While the anthocyanins present in berries are mainly monoglycosyl derivatives [28], potatoes contain two or more glycosyl groups and acyl-glycosyl conjugates [3,4], which contribute to the higher stability of this phenolic compound. Similarly, in a study of isolated anthocyanins, Zhao et al. [29] observed that anthocyanins with a large number of sugar moieties in phenolic compounds have complex structures and high stability. Little information is available on stability studies of HCADs. Mozetic et al. [30] conducted a study of sweet cherries treated with 1-methylcyclopropene, a plant-growth regulator responsible for prolonging the shelf life of different fruits, and reported high stability in HCADs, with an average decrease of only 22% after twelve days of cold storage. Studies by Brownmiller et al. [31] on blueberries stored for 6 months under different processes (canned in syrup, canned in water, pureed, clarified juice and unclarified juice) revealed a significant decrease in procyanidin content during the 6-month storage period. Only 8% and 11% of procyanidins were retained in the clarified and unclarified juices, respectively; 7% remained in the purees; and 22% and 32% were found in blueberries canned in syrup and canned in water, respectively. These results are similar to those obtained in our study, in which the final concentration of phenolic compounds showed a drastic decrease during storage. With respect to the stability of phenolic compounds, our results differ from those in the literature. Ercoli et al. [18] conducted a study on colored potato solid extracts, and the authors observed a 10% decrease in total phenol concentration after four months of storage. In a study by Tereucan et al. [22] on milk and yogurt samples colored with natural dye from colored-flesh potatoes, a decrease of between 53% and 69% in total phenolic concentration was reported in the second week; however, between the second and eighth weeks, the concentration was stable in milk samples. In contrast, in yogurt samples, only a 36% decrease was detected. For other food sources, similar results regarding the low stability of phenolic compounds over time have been reported. For example, the phenolic content of extra virgin olive oil was assessed after the oil was stored for 1 year in various types of containers (clear glass, dark glass and polyethylene) under dark or light conditions. Exposure to light during storage led to a reduction in the total phenolic content in all cultivars studied, particularly in those stored in polyethylene containers. On average, there was a decrease of 30.5% under dark conditions, while oil stored in containers exposed to light experienced a reduction of approximately 60% [32]. These results align with other studies that have reported on qualitative and quantitative shifts in phenolic content during storage as a result of decomposition and oxidation reactions [32,33,34].

The high response detected with the CUPRAC method may be attributed to the high concentration of HCADs in the samples. Ruiz et al. [35] demonstrated that samples with high HCAD or flavonol content showed high CUPRAC levels. With this method, the values were higher than the levels in colored-flesh potato tubers reported by Ercoli et al. [4], in which the highest value obtained was under 2 μmol g^−1^. The results obtained with the TEAC method were low compared with studies performed by Ruiz et al. [3] on colored potato chips, reaching levels between 2.3 and 8.9 μmol g^−1^. However, in the same study by Ruiz et al. [3], higher levels of anthocyanins and HCADs were reported compared with our results. With the DPPH method, our activity results were lower than those of similar studies in which the antioxidant activity was evaluated using the DPPH method in deep-fried purple potatoes, obtaining values varying from 38.9 μmol g^−1^ dry weight (DW) to 33.2 μmol g^−1^ DW [36]. ORAC is an assay based on a hydrogen atom transfer (HAT) reaction; it is the only method that completes the action of free radicals and uses an area under the curve (AUC) technique for quantification [37]. There is little information available on the antioxidant activity of colored-flesh potatoes as determined with the ORAC method. López-Cobo et al. [38] performed a study on two genotypes of yellow-flesh potatoes (Blue Bell and Melody) and showed that the antioxidant activity analyzed with the ORAC method presented values of 18.9 μmol 100 g^−1^ DW and 24 μmol 100 g^−1^ DW, respectively. These results were lower relative to the values obtained in our study on colored-flesh potato chips.

For antioxidant activity, our results, based on the CUPRAC method, revealed a 45.23% decrease relative to the initial measurement and showed higher stabilities compared with those achieved in studies on colored-flesh potato extracts, in which researchers observed an 81% decrease, on average, in antioxidant activity under acidic conditions (pH 1, 2 and 3) [18]. The activity determined using the TEAC method was more stable (a 52.94% decrease after twenty-four weeks) than that found in the abovementioned study (a 77% decrease after four months) and relative to results from a study on milk and yogurt samples containing a natural dye from colored-flesh potatoes, in which researchers showed an average 74% decrease at week 8 [18,22]. With the DPPH method, the highest stability in antioxidant activity was observed during storage, with a 39.75% decrease with respect to the initial measurement. This indicated greater stability than that observed in another colored-flesh potato extract study, with a 65% decrease after storage. Furthermore, in a study on milk and yogurt samples, decreases of 92–98% in milk samples and 78–95% in yogurt samples were observed during the first three weeks, followed by a slow decrease toward the end of the study [18,22]. The oxygen radical absorbance capacity (ORAC) assay was developed to measure the antioxidant capacity of foods [39], but there is no information regarding stability studies using this method. The variety of values obtained using each of these methodologies may arise from the fact that these methods evaluate different compounds. It is remarkable that, in our previous reports, we demonstrated that different antioxidant methods can respond to different families of compounds. For example, the TEAC method responds better to anthocyanins, while the CUPRAC and DPPH methods respond better to non-colored compounds such as hydroxycinnamic acids and flavonols [3,35]. Regarding the ORAC method, it was added to this study as a standard method to determine food quality because this method was developed to assess antioxidant capacity in foods [39].

Color is one of the key factors that affect the quality of food products because some products attract consumer attention because of their appearance [40]. Regarding color composition, the percentage of yellow was predominant throughout the whole study. This predominance can be clearly seen in the potato chip samples, with a slight red/pink and purple tonality in comparison with the high presence of yellow. These results vary in comparison with a study on natural dyes from colored-flesh potatoes by Tereucán et al. [22], in which the predominant color percentage was red, followed by yellow and blue in both milk and yogurt samples. A direct correlation between the total anthocyanins and the color intensity was observed. Studies on red raspberry juice by Chen et al. [27] indicated that the loss of color quality during storage is related to anthocyanin degradation, which is similar to the results obtained in our analyses.

Interestingly, although a relationship between anthocyanin degradation and loss of color quality was mentioned, in our study, no change in the color of the potato chips could be seen despite the decrease in anthocyanin concentrations. Studies on color stability in different food products, e.g., jam or yogurt made from berries, have shown that the rate of color loss is slower than the rate of anthocyanin degradation, and the presence of other compounds can have a considerable effect on color [41,42]. The results presented in the works of Vukoja et al. [43] are similar to our findings, as they studied the stability of anthocyanins in three types of cherry jams (light, regular and extra) after 8 months of storage and observed a decrease in these compounds in all the jams. In contrast, the color percentage increased to varying degrees after storage in all three types of jams. This behavior can be explained by the formation of anthocyanin polymers in response to storage, a result that has been observed in other studies showing that an increase in color percentage occurs alongside a loss in anthocyanin content [44,45].

In general, the phenolic compounds identified in this study decreased drastically during the storage period, which directly correlated with a decrease in the antioxidant activity of these compounds. Tereucán et al. [22] presented a high correlation between anthocyanins and HCADs observed in yogurt during the first week of analysis, similar to the results of our study. The correlation between total phenolic compounds and antioxidant activity was due to the high presence of anthocyanins and HCADs during the initial week of storage, which was mostly demonstrated by the CUPRAC and TEAC methods [3,24]. On the other hand, in the final week of the study, hue and yellow coloration were predominant, similar to what was observed by Tereucán et al. [22] in milk stored at room temperature, which was attributed to a loss of color in their samples due to the reduced stability of phenolic compounds. These compounds have been widely studied in the food industry because of their undeniable beneficial effects when regularly consumed in the diet. Another study on sweet potatoes showed that the concentrations of phenolic compounds can also vary depending on the cooking technique. Roasting sweet potatoes in the oven resulted in an increase in the levels of caffeoylquinic acids, while prolonged cooking times in water led to a decrease in the same compounds [46]. These results indicate that variables such as the origin, processing and storage of colored potato chips can directly influence the phenolic compound content in them. These processes, in turn, depend on other factors, such as the cooking method, temperature and time. For this reason, it is crucial to conduct these types of studies, which allow us to improve the processes for potentially obtaining more nutritious foods for the human diet.

## 4. Materials and Methods

### 4.1. Reagents

Standard petunidin-3-glucoside (98.95%) was obtained from PhytoLab (Vestenbergsgreuth, Germany). Trolox (6-hydroxy-2,5,7,8-tetramethylchroman-2-carboxylic acid) (97%), ABTS (2,2′-azino-bis(3-ethylbenzothiazoline-6-sulfonic acid)) (>98%), gallic acid (97.5–102.5% titration), chlorogenic acid (≥95%), neocuproin (≥98%), DPPH (2,2-diphenyl-1-picrylhydrazyl), Folin–Ciocalteu reagent, AAPH (2,2′-azobis-2-methyl-propanimidamine, dihydrochloride) (97%), fluorescein (95%), monobasic potassium phosphate (KH_2_PO_4_), dibasic potassium phosphate (K_2_HPO_4_), potassium persulfate (K_2_O_8_S_2_), ammonium acetate and copper (II) chloride (CuCl_2_) were obtained from Sigma-Aldrich (Steinheim, Germany). Water (HPLC grade), ethanol (HPLC grade), methanol (HPLC grade), acetonitrile (HPLC grade), formic acid (p.a. grade) and sodium carbonate (K_2_O_8_S_2_) were obtained from Merck (Darmstadt, Germany).

### 4.2. Samples

**Figure 5:**
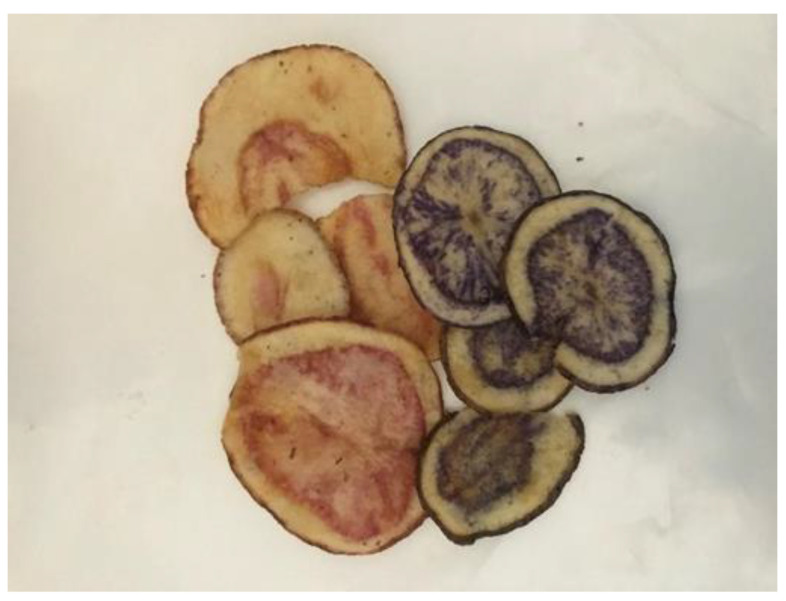
Samples of colored-flesh potato chips.

Potato chips (“PatPot Chips”) were provided by C-M SpA (Puerto Varas, Chile) (Figure 5). Colored-flesh potato chips were stored in their original bags at room temperature in the dark until analysis. Nutritional value assessments were carried out using one bag per week for a period of two months and then every fifteen days. All experiments were carried out with three replicates, and the results represent the mean values.

### 4.3. Extraction of Anthocyanins and HCAD

Five grams of potato chips was smashed and mixed with 10 mL of extraction solvent (methanol:formic acid, 97:3 *v*/*v*) in a test tube protected from light. Subsequently, the samples were sonicated with an ultrasonic processor at 130 Watt (Sonics & Materials, Newtown, CT, USA) for 60 s at 40% amplitude, shaken for 10 min at 200× *g* and centrifuged (Lab companion, Seoul, Republic of Korea) for 10 min at 3000× *g*. Finally, the supernatant was recovered and stored at −20 °C until analysis.

### 4.4. Identification and Quantification of Anthocyanins and HCADs

The chromatographic analyses were performed according to Ruiz et al. [3]. The determination of anthocyanins and HCADs was performed using high-performance liquid chromatography with diode array detection (HPLC-DAD) (Shimadzu, Tokyo, Japan) equipped with a quaternary pump (LC-20AT), a degassed unit (DGU-20A5R), a column oven (CTO-20A), an autosampler (SIL-20A) and a UV-Vis diode array detector (SPD-M20A). Compounds were separated using a C_18_ column (Kromasil: 250 × 4.6 mm, 5 µm) protected with a C_18_ precolumn (NovaPak, Waters, Milford, MA, USA, 22 × 3.9 mm, 4 µm) at 40 °C. The mobile phases were A) water:acetonitrile:formic acid 87:3:10 (*v*:*v*:*v*) and B) water:acetonitrile:formic acid 40:50:10 (*v*:*v*:*v*). The mobile phase B gradient was from 6% to 30% over 15 min; from 30% to 50% over 15 min; from 50% to 60% over 5 min; and from 60% to 6% over 6 min, followed by stabilization for 10 min at a flow rate of 0.8 mL min^−1^. The injection volume was 20 μL. Anthocyanins and HCADs were quantified at 520 nm and 320 nm, respectively, using petunidin-3-glucoside and chlorogenic acid as external calibration standards (Table 3). Identity assignments were performed according to Nova et al. [47] using an HPLC-DAD-QTOF-MS/MS compact (Bruker Daltonics GmbH, Bremen, Germany). Instrument control and data collection were carried out using Compass DataAnalysis 4.4 SR1 (Bruker Daltonics GmbH), using negative ionization mode for HCADs and positive ionization mode by anthocyanins.

### 4.5. Determination of Total Phenols by the Folin–Ciocalteu Method

Total phenol concentrations were determined using the Folin–Ciocalteu method as described by Singleton et al. [48], with minor modifications. The reagents were added in the following order to a 1.5 mL Eppendorf tube: 15 µL of standard or extract, 750 µL of deionized water, 75 µL of Folin–Ciocalteu reagent, 300 µL of sodium carbonate 20% *w*/*v*, 360 µL of deionized water. The solutions were shaken and incubated at 20 °C for 30 min in the dark. Finally, 250 µL of the solution was added to a 96-well plate, and the absorbance was read at 750 nm in a UV-Vis Epoch microplate spectrophotometer (BioTek, Winooski, VT, USA). The tests were performed using gallic acid as a standard (Table 3), in which the concentrations in the calibration curve corresponded to 100, 200, 300, 400 and 500 mg L^−1^.

### 4.6. Determination of Antioxidant Activity

For the determination of Trolox equivalent antioxidant capacity (TEAC), solutions must be adjusted to an absorbance of 0.70 ± 0.05 (734 nm) via dilution with ethanol or water before use [49]. Briefly, 245 µL of ABTS^+^ was added to 96-well plates, and the first reading was performed. Subsequently, 5 µL of Trolox or the sample to be analyzed was added to each well and incubated for 30 min at 30 °C in the dark under gentle shaking (+/− 30 rpm), and finally, the second reading was performed. The measurements were made using a UV-Vis Epoch microplate spectrophotometer (BioTek, Winooski, VT, USA), and the results were expressed as µmol Trolox equivalents g^−1^.

For cupric reducing antioxidant capacity (CUPRAC), the following reagents were added to a 96-well microplate: 50 µL of 10 mM CuCl_2_, 50 µL of 7.5 mM neocuproine and 50 µL of 1 M ammonium acetate buffer (pH 7), and the mixture was incubated in the dark at 27 °C for 15 min. Next, 100 µL of Trolox standard or sample was added, and the solution was incubated for 30 min at 27 °C. Quantification was carried out at 450 nm using a UV-Vis Epoch microplate spectrophotometer (BioTek, Winooski, VT, USA), and the results were expressed as µmol Trolox equivalents g^−1^ [50].

The determination of antioxidant activity using DPPH was based on a methodology described by Maldonado et al. [51], with some modifications. Briefly, 240 µL of 0.1 mM DPPH radical dissolved in ethanol was added to a 96-well plate, and the first absorbance reading was collected. Then, 10 µL of the Trolox curve or sample was added; the solution was incubated for 30 min in the dark; and the second absorbance reading was taken. Measurements were performed at 517 nm using a UV-Vis Epoch microplate spectrophotometer (BioTek, Winooski, VT, USA), and the results were expressed as µmol Trolox equivalents g^−1^.

Determination of oxygen radical absorbance capacity (ORAC) was performed following a methodology described by Cao et al. [52]. In brief, 200 µL of water was added to a 96-well microplate fluorometer. Next, 25 µL of Trolox standard/sample was mixed with 150 µL of fluorescein (11.12 × 10^−2^ µM), and the solution was incubated for 30 min at 37 °C before adding 25 µL of AAPH solution. Fluorescence was measured every 5 min for 90 min in a Synergy HTX Multi-Mode reader (BioTek, Winooski, VT, USA), and the values were expressed as µmol Trolox equivalents g^−1^.

### 4.7. Color Determinations

Color determinations were carried out using the CIELab method [53], in which absorbance values are measured at three different wavelengths (420, 520 and 620 nm), and the following parameters were determined through mathematical models: color intensity (CI = A_420_ + A_520_ + A_620_), color tonality (To = A_420_/A_520_), percentage of yellow (%Ye = A_420_ × 100/CI), percentage of red (%Red = A_520_ × 100/CI), percentage of blue (%Bl = A_620_ × 100/CI), redness a = 3.282 − (12,142 × A_420_) + (74.509 × A_520_) − (81.731 × A_620_), yellowness b = −0.716 + (91.261 × A_420_) − (41.675 × A_520_) − (54.352 × A_620_), lightness L = 98.172 − (17.497 × A_420_) − (35.817 × A_520_) − (10.416 × A_620_), saturation C = 3.035 − (14.582 × A_420_) + (59.488 × A_520_) − (91.112 × A_620_) and hue angle h = 9.569 + (299.84 × A_420_) − (186.894 × A_520_) − (93.708 × A_620_). Determinations were carried out in a 1 mm quartz cuvette using a Genesys 10 s UV-Vis spectrometer (Thermo Scientific, Waltham, MA, USA).

### 4.8. Statistical Analysis

All the statistical analyses and calculations were conducted using R version 4.2.1. One-way ANOVA was employed to examine significant variances between the measurements of each experimental variable. For the variables that exhibited significant differences, the means were compared using Tukey’s HSD test along with the appropriate post hoc analysis with “agricolae” v. 1.3.5 package. Furthermore, the datasets corresponding to weeks 0, 4, 8, 13, 17, 21 and 23 were subjected to principal component analysis (PCA). Confidence ellipses representing the group means for each week were generated using the “FactoMineR” v. 2.7 and “factoextra” v. 1.0.7 packages.

## 5. Conclusions

The results obtained in this study indicate that chips made from colored-flesh potatoes are a good source of phenolic compounds, mainly HCADs, which remained stable during a storage period of twenty-four weeks. Although colored potatoes are rich in anthocyanins, they do not constitute a stable food matrix for anthocyanins when cooked as chips since the anthocyanins were almost completely degraded after 23 weeks of storage. Similar trends were observed in the total phenolic compound content and antioxidant activity using all methodologies, with a slight decrease over time. The color index did not show large differences in relation to stability. Potato chips made from colored-flesh potatoes might be an optimal alternative snack for global consumption given their high phenolic compound content, including anthocyanins and HCADs, which possess high antioxidant activity and other potential benefits for human health.

## Figures and Tables

**Table 1:** Identification of phenolic compounds as anthocyanins and hydroxycinnamic acid derivatives (HCADs) in colored potato chips using HPLC-DAD-ESI-MS/MS.

Peak Number	t_R_ (min)	Tentative Identification	[M]^+^	[M − H]^−^	Product Ions	^max (nm)
1	8.2	5-caffeoylquinic acid	-	353.1	191.1	325
2	9.1	n.i	-	-	-	323
3	10.5	Caffeoylquinic acid isomer	-	353.1	191.1	319
4	19.5	Petunidin-3-p-coumaroylrutinoside-5-glucoside	933.2	-	771.2; 479.0; 317.0	530
5	21.0	Petunidin-3-feruloylrutinoside-5-glucoside	963.3	-	479.0; 317.0	-
6	21.5	Pelargonidin-3-p-coumaroylrutinoside-5-glucoside	887.3	-	271.3; 725.2; 433.1	504
7	22.0	Peonidin-3-p-coumaroylrutinoside-5-glucoside	917.1	-	755.3; 463.2; 301.2	528
8	22.5	n.i		-	-	504

**Table 2:** Individual phenolic compound concentrations (mg kg^−1^) in colored-flesh potato chips identified using HPLC-DAD. nd: undetected compounds, HCADs: hydroxycinnamic acid derivatives. Identifications according to Table 1: peak 1: 5-caffeoylquinic acid; peak 2: unidentified; peak 3: caffeoylquinic acid isomer; peak 4: petunidin-3-p-coumaroylrutinoside-5-glucoside; peak 5: petunidin-3-feruloylrutinoside-5-glucoside; peak 6: pelargonidin-3-p-coumaroylrutinoside-5-glucoside; peak 7: peonidin-3-p-coumaroylrutinoside-5-glucoside; peak 8: unidentified. Different letters indicate significant differences according to Tukey’s test (*p* < 0.05). Results are given as the mean of the values for each treatment ± the standard error.

	HPLC, HCADs (mg kg^−1^)			HPLC, Anthocyanins (mg kg^−1^)				
Week	Peak 1	Peak 2	Peak 3	Peak 4	Peak 5	Peak 6	Peak 7	Peak 8
1	97.82 ± 34.47 ^a^	nd	1.79 ± 0.54 ^ab^	nd	3.24 ± 0.94 ^a^	1.61 ± 1.56 ^ab^	nd	nd
2	52.47 ± 8.42 ^bcd^	nd	1.24 ± 1.07 ^bc^	1.04 ± 0.91 ^bc^	0.46 ± 0.80 ^c^	nd	nd	nd
3	40.27 ± 8.96 ^cd^	nd	0.,47 ± 0.81 ^bc^	1.62 ± 1.41 ^ab^	nd	nd	nd	nd
4	51.6 ± 20.98 ^bcd^	nd	0.93 ± 0.81 ^bc^	1.94 ± 0.47 ^ab^	nd	nd	nd	nd
5	46.24 ± 6.58 ^bcd^	nd	1.00 ± 0.11 ^bc^	1.98 ± 0.07 ^ab^	nd	nd	nd	nd
6	54.12 ± 5.65 ^bcd^	nd	nd	2.14 ± 0.29 ^ab^	nd	nd	nd	nd
7	69.93 ± 9.85 ^abc^	nd	nd	2.10 ± 0.22 ^ab^	nd	nd	nd	nd
8	39.21 ± 8.32 ^cd^	nd	nd	1.53 ± 0.07 ^ab^	nd	nd	nd	nd
9	45.83 ± 6.49 ^bcd^	nd	1.20 ± 1.04 ^bc^	1.73 ± 0.26 ^ab^	nd	nd	nd	nd
12	21.20 ± 1.12 ^d^	23.02 ± 0.48 ^a^	nd	nd	1.52 ± 0.14 ^b^	nd	nd	nd
14	25.08 ± 3.75 ^d^	5.20 ± 0.78 ^b^	nd	nd	nd	0.69 ± 0.62 ^ab^	nd	nd
16	23.13 ± 3.38 ^d^	4.30 ± 0.30 ^b^	nd	nd	nd	1.17 ± 0.24 ^ab^	0.28 ± 0.48 ^b^	nd
18	39.20 ± 9.74 ^cd^	5.63 ± 2.14 ^b^	1.89 ± 0.49 ^ab^	nd	nd	1.89 ± 1.18 ^a^	1.21 ± 0.24 ^a^	0.34 ± 0.59 ^a^
20	35.88 ± 14.78 ^cd^	5.37 ± 2.06 ^b^	1.89 ± 0.60 ^ab^	nd	nd	1.20 ± 1.10 ^ab^	0.42 ± 0.74 ^b^	nd
22	36.87 ± 5.93 ^cd^	5.19 ± 0.63 ^b^	1.86 ± 0.20 ^ab^	nd	2.14 ± 0.28 ^b^	nd	nd	nd
24	26.08 ± 8.20 ^d^	4.02 ± 1.07 ^b^	1.33 ± 0.28 ^bc^	nd	nd	nd	nd	nd

**Table 3:** Analytical parameters for chromatographic and spectrophotometric methods.

Method	Standard	Equation	R^2^	DL	QL	LR	CV (%)
HPLC	Petunidin-3-glucoside; Chlorogenic acid	y = 55,324x − 44,878; y = 73,284x + 6553.5	0.998; 1.000	0.332 mg L^−1^; 0.042 mg L^−1^	1.108 mg L^−1^; 0.140 mg L^−1^	1.108–100 mg L^−1^; 0.140–100 mg L^−1^	0.32%; 0.39%
Folin	Gallic acid	y = 0.0008x + 0.0492	0.999	4.535 mg L^−1^	15.116 mg L^−1^	15.116–100 mg L^−1^	2.41%
CUPRAC	Trolox	y = 3.158x + 0.1822	0.993	0.032 mmol L^−1^	0.105 mmol L^−1^	0.105–0.7 mmol L^−1^	4.28%
TEAC	Trolox	y = 0.4249x + 0.0193	0.998	0.022 mmol L^−1^	0.074 mmol L^−1^	0.074–0.7 mmol L^−1^	13.35%
DPPH	Trolox	y = 0.4031x + 0.0769	0.996	0.011 mmol L^−1^	0.037 mmol L^−1^	0.037–0.7 mmol L^−1^	4.49%
ORAC	Trolox	y = 0.4296x + 4.2862	0.995	0.532 umol L^−1^	1.774 umol L^−1^	1.774–80 umol L^−1^	13.96%

DL, detection limit; QL, quantification limit; LR, linear range; CV%, coefficient of variation.

## Data Availability

The data presented in this study are available upon request from the corresponding author.

## Supplementary Materials

The following are available online https://www.mdpi.com/article/10.3390/molecules28166047/s1: Figure S1: HPLC-DAD chromatogram of phenolic compounds in *Solanum tuberosum* chips: (A) hydroxycinnamic acids at 320 nm and (B) anthocyanins at 520 nm. Figure S2: Mass spectrometry (MS/MS) spectra under negative ionization, (A,B) with hydroxycinnamic acids and under positive ionization (C–F) with anthocyanins in *Solanum tuberosum* chips. (A) peak 1, 5-caffeoylquinic acid; (B) peak 3, caffeoylquinic acid isomer; (C) peak 4, petunidin-3-p-coumaroylrutinoside-5-glucoside; (D) peak 5, petunidin-3-feruloylrutinoside-5-glucoside; (E) peak 6, pelargonidin-3-p-coumaroylrutinoside-5-glucoside; and (F) peak 7, peonidin-3-p-coumaroylrutinoside-5-glucoside.

## Sample Availability

Samples of the compounds are available from the authors.
