# Supplementary material for: Stability of Phenolic Compounds, Antioxidant Activity and Color Parameters in Colored-Flesh Potato Chips
Source: Molecules. 2023 Aug 14;28(16):6047. doi: 10.3390/molecules28166047 (PMC10459281; doi:10.3390/molecules28166047)
Supplement: Supplementary file 1 [file molecules-28-06047-s001.zip › molecules-2512463-supplementary.pdf]

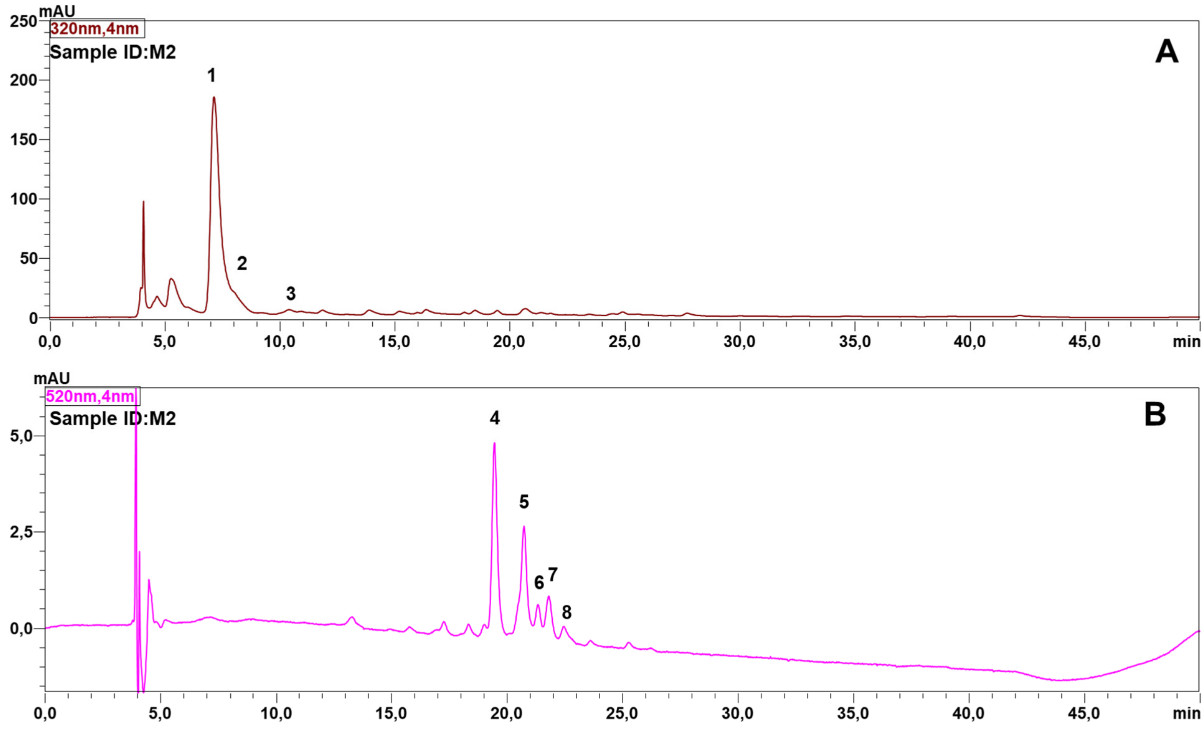

Figure S1: HPLC-DAD chromatogram of phenolic compound in *Solanum tuberosum* chips. Where A) hydroxycinnamic acids at 320 nm and B) anthocyanins at 520 nm

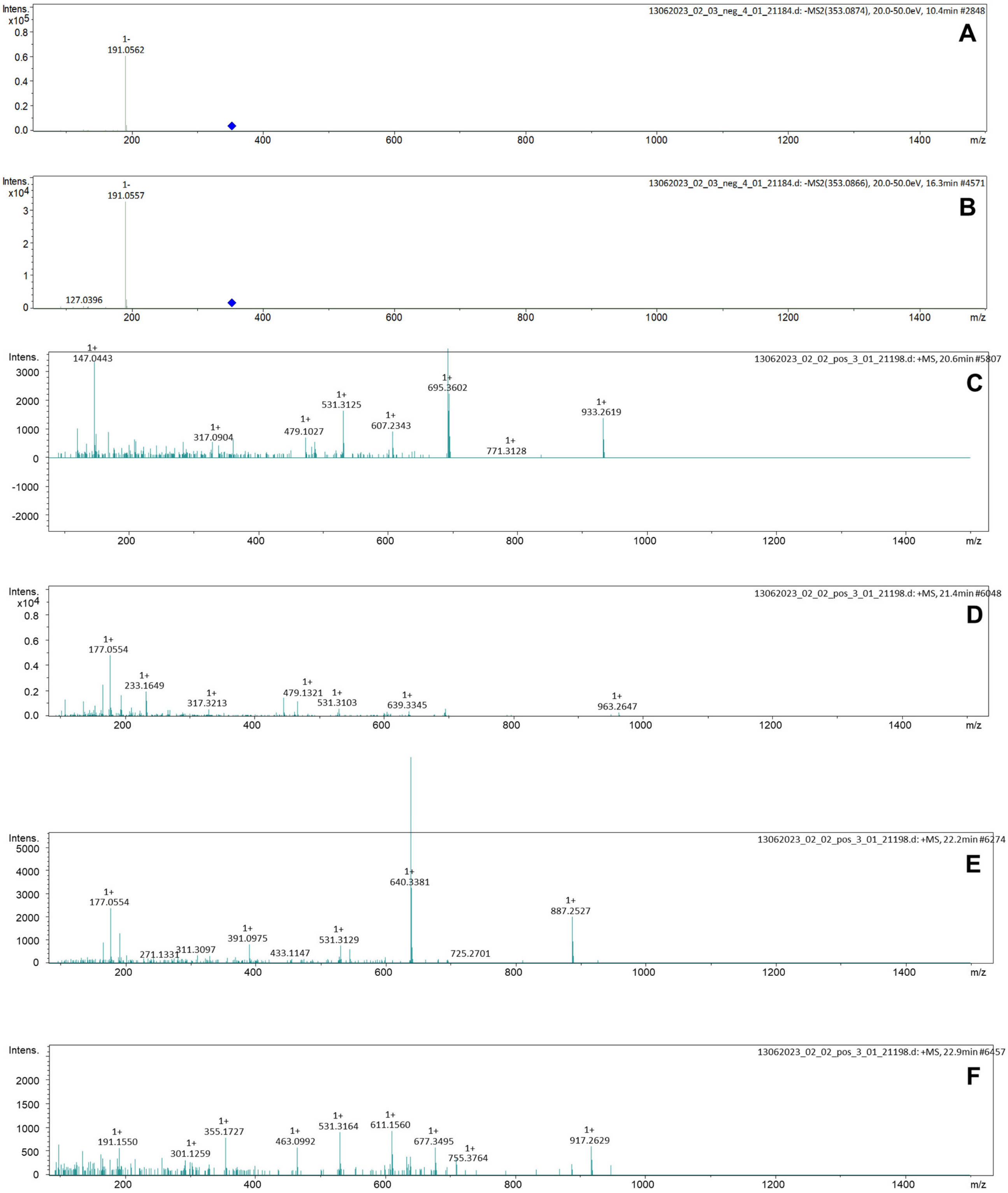

Figure S2: Mass spectrometry (MS/MS) spectra under negative ionization (A,B) by hydroxycinnamic acids and under positive ionization (C-F) by anthocyanins in *Solanum tuberosum* chips. Where A) peak 1, 5-caffeoylquinic acid; B) peak 3, caffeoylquinic acid

isomer; C) peak 4, petunidin-3-p-coumaroylrutinoside-5-glucoside; D) peak 5, petunidin-3-feruloylrutinoside-5-glucoside; E) peak 6, pelargonidin-3-p-coumaroylrutinoside-5-glucoside; and F) peak 7, peonidin-3-p-coumaroylrutinoside-5-glucoside.
